# Prenatal Hypoxic-Ischemic Insult Changes the Distribution and Number of NADPH-Diaphorase Cells in the Cerebellum

**DOI:** 10.1371/journal.pone.0035786

**Published:** 2012-04-23

**Authors:** Tiago Savignon, Everton Costa, Frank Tenorio, Alex C. Manhães, Penha C. Barradas

**Affiliations:** 1 Departamento de Farmacologia e Psicobiologia, Instituto de Biologia Roberto Alcantara Gomes, Universidade do Estado do Rio de Janeiro, Rio de Janeiro, Brazil; 2 Departamento de Ciências Fisiológicas, Instituto de Biologia Roberto Alcantara Gomes, Universidade do Estado do Rio de Janeiro, Rio de Janeiro, Brazil; Tokyo Medical and Dental University, Japan

## Abstract

Astrogliosis, oligodendroglial death and motor deficits have been observed in the offspring of female rats that had their uterine arteries clamped at the 18^th^ gestational day. Since nitric oxide has important roles in several inflammatory and developmental events, here we evaluated NADPH-diaphorase (NADPH-d) distribution in the cerebellum of rats submitted to this hypoxia-ischemia (HI) model. At postnatal (P) day 9, Purkinje cells of SHAM and non-manipulated (NM) animals showed NADPH-d+ labeling both in the cell body and dendritic arborization in folia 1 to 8, while HI animals presented a weaker labeling in both cellular structures. NADPH-d+ labeling in the molecular (ML), and in both the external and internal granular layer, was unaffected by HI at this age. At P23, labeling in Purkinje cells was absent in all three groups. Ectopic NADPH-d+ cells in the ML of folia 1 to 4 and folium 10 were present exclusively in HI animals. This labeling pattern was maintained up to P90 in folium 10. In the cerebellar white matter (WM), at P9 and P23, microglial (ED1+) NADPH-d+ cells, were observed in all groups. At P23, only HI animals presented NADPH-d labeling in the cell body and processes of reactive astrocytes (GFAP+). At P9 and P23, the number of NADPH-d+ cells in the WM was higher in HI animals than in SHAM and NM ones. At P45 and at P90 no NADPH-d+ cells were observed in the WM of the three groups. Our results indicate that HI insults lead to long-lasting alterations in nitric oxide synthase expression in the cerebellum. Such alterations in cerebellar differentiation might explain, at least in part, the motor deficits that are commonly observed in this model.

## Introduction

Systemic perinatal insults alter brain development and lead to cerebral palsy, cognitive impairment and epilepsy in children. Despite advances in perinatal medicine, the proportion of children with chronic neurologic deficits after perinatal injury has remained stable [1]. Human infant brains show oligodendrocyte loss, hypomyelination, astrogliosis [2], and perturbed cortical development [3] after perinatal insults. The mechanisms underlying these pathological changes remain, thus far, largely unclear.

Because various insults at different gestational stages induce elevated levels of cytokines and disrupt brain development, it has been proposed that aberrant cytokine expression underlies perinatal brain injury [4], [5]. The pathogenesis of perinatal brain insults is, however, likely to involve numerous pathways associated with cytokines and oxygen-free radical species [6], [7] but their relative contributions have yet to be defined.

Nitric oxide (NO) has important roles in both inflammatory and developmental events and it has been described as presenting both neuroprotective and neurotoxic properties [8]. NO is synthesized from L-arginine by isoforms of the nitric oxide synthase (NOS) enzyme family: neuronal (nNOS) and endothelial (eNOS), which are constitutively expressed, and inducible NOS (iNOS), which is expressed under proper stimulus, as inflammation or trauma. All NOS isoforms use nicotinamide adenine dinucleotide phosphate (NADPH) as an electron donor and may be identified by NADPH-diaphorase (NADPHd) histochemistry [9]. Hope and colleagues, using affinity chromatography followed by anion-exchange HPLC, showed that nitric oxide synthase and NADPH diaphorase activity copurified yielding a band of 150 kDa on SDS/PAGE, which corresponds to the size of the NOS purified from the rat cerebellum [10]. In paraformaldehyde-fixed tissues, NADPH-d reduces nitroblue tetrazolium to a stable, insoluble dark-blue product, formazan [11].

NO has been implicated in neural proliferation and migration as well as in synaptic formation and maturation. Neuronal NOS is expressed in the brain during development and is considered to be an important factor in maturational processes. It has been shown that nNOS is expressed in cortical plate neurons at embryonic day (E) 15 and decreases posnatally [12]. Other authors [13]–[16], using in situ hybridization or hystochemistry for NADPH-diaphorase, suggested the involvement of NO in embryonic and postnatal development. NO plays a leading role in refining axonal connectivity [17]–[23] and is also involved in cellular migration [24], [25]. Cerebellar cell populations expressing NOS during development consist of Purkinje neurons, granule neurons after migration from the external granular layer (EGL) to the internal granular layer (IGL), and glial cells, represented by microglia and astrocytes. In adult animals, NOS expressing cells are represented by mature granule neurons as well as by the molecular layer interneurons, such as basket and stellate cells, giving rise to a very strong stain of both the granular (GL) and the molecular layers (ML)(see [26] for review). Several authors have reported that there is a differential timing regarding folial development [27] as well as NOS expression during development [28], [29].

Enhancement of all NOS isoforms expression has been reported in the retina and other central nervous system (CNS) areas in response to hypoxia [30], [31]. NO produced by eNOS has a protective response after hypoxic-ischemic episodes causing vasodilation, which leads to an increase in blood flow [32]. However, it has been proposed that, besides its beneficial effects, producing vasodilation and increased blood flow, eNOS is also involved in vascular endothelial growth factor (VEGF) induced vascular hyperpermeability [33], which contributes to disruption of the blood-brain barrier, resulting in vascular leakage [34], [35]. NO becomes noxious if it is produced in excess [36] and its overproduction contributes to excitotoxicity, resulting in cell death and axonal damage. Glial cells have been suggested as the major source of this NO overproduction [37], [38]. Levels of iNOS in the CNS are low, but iNOS can be induced in astrocytes or microglial cells following events such as inflammation, viral infection or trauma [9], [39].

Robinson and colleagues [40] hypothesized that systemic insults in infants born prematurely initiate a cascade of harmful mediators that disrupt glia and neurons in both the white and gray matter and that such changes are likely to result from both direct effects on maturing neural precursors, and environmental alterations in which neural development is progressing. Since a significant component of perinatal brain damage in humans results from preterm systemic insults, they proposed a rodent model of prenatal systemic HI insult, in which the four uterine arteries of pregnant rats at the 18th gestation day were clamped. Using this model resulted in white matter astrogliosis, oligodendrocyte loss and axonal disruption in both white matter and cortex in the offspring, during development and at adulthood. These alterations in brain formation are similar to those that have been observed in human samples [2], [3]. The HI insult also caused delays in motor development in young adult rats [40]. Despite the fact that rats do not develop cerebral palsy, even when severe injuries are present, this systemic rodent prenatal HI insult may model human perinatal brain injury in several important ways, including functional association of altered brain development with motor delay, and consequently provides novel insights into the pathogenesis of human perinatal brain insults.

The cerebellum has a major role in motor control [41]–[43], and several injuries in the cerebellum were shown in extremely low birth weight children with clinical diagnosis of cerebral palsy [44]. Furthermore, in another rodent model of maternal hypoxia it has been shown that Purkinje cells in the cerebellum are injured leading to a delay in motor reflex [45]. Thus, in the current study, we used a modified model of systemic HI in rats at E18 as described by Robinson and colleagues [40] to address how intrauterine insults affect NOS distribution during postnatal cerebellar development.

## Materials and Methods

### Rat prenatal hypoxic–ischemic insult

All animal studies were conducted in accordance with the principles and procedures approved by the university's Animal Care Committee (CEA/019/2010) and National Institute of Health Guide for the Care and Use of Laboratory Animals. All efforts were made to minimize the number of animals used and their suffering. All animals that have been used were from our colony. Pregnant Wistar rats in the 18^th^ gestation day were anesthetized with Avertin® (tribromoethanol, 0.3 mg/Kg i.p.) in fractioned doses. A midline laparotomy was performed, the uterine horns were exposed and a haemostatic forceps was used on each the four uterine arteries (HI group). Sham controls had the uterine horns exposed, but no arteries were clamped (SHAM group). The forceps were left in place clamping the arteries for 45 min, during which time the uterine horns were kept moist. A clamping period of 45 minutes was chosen because it is the minimum period of time that results in most of the morphological changes that were reported in previous papers [40], [46], [47]. After this period, the haemostatic forceps were removed, the uterine horns replaced in the abdominal cavity and the abdominal wound sutured in layers. A third group of non-manipulated pregnant rats were used (NM group). Sodium dipirone solution (10 mg/100 g of body weight, i.p.) was administered for post-operative pain relief. Rats were awakened from anesthesia, monitored to ensure adequate recovery, and returned to the animal facility. Pups were born at term (E22) with no surgical intervention. The HI animals did not show any gross abnormalities during the neonatal period. Each litter had a minimum of 6 and a maximum of 8 animals, and a minimum of 5 animals in each age from at least 3 different litters per group was used for the histochemistry and immunohistochemistry procedures.

### NADPH-d histochemistry

At postnatal ages P9, P23, P45 and P90, animals were anesthetized with sodium pentobarbital (50 mg/kg) and intracardially perfused with 0.9% saline solution followed by 4% paraformaldehyde in 100 mM phosphate buffer (pH 7.4) and then by the same fixative plus 10% sucrose for cryoprotection. After dissection, the cerebella were immersed in 100 mM phosphate buffer containing 20% sucrose at 4°C and, on the following day, they were parasagittally sectioned in the vermis region (0.5 mm mediolateral distance) at 40 µm (P9) or at 60 µm (P23, P45 and P90) on a cryotome (SLEE) at −20°C. Sections were collected in 50 mM Tris buffer (pH 7.4), washed twice in this buffer and submitted to a NADPH-d histochemistry technique as previously described [10], [48]. In brief, free-floating sections were incubated at 37°C for 60 min in 0.05 M Tris-HCl buffer (pH7.4) containing 0.3% Triton X-100, 0.4 mg/mL nitroblue tetrazolium (Sigma, MO, USA), and 0.8 mg/mL β-NADPH (Sigma). Sections were washed three times with 0.05 M Tris-HCl (pH7.4) and mounted on gelatin-coated slides. The slides were air dried overnight, dehydrated in crescent alcohol solutions, clarified in xilol and mounted using Entellan as mounting medium. Negative control sections were incubated without the addition of β-NADPH to the reaction solution. Furthermore, neurons that express NADPH-d in pontine tegmental nuclei [10] were used as positive controls of hystochemical reaction. Additional sections were stained with haematoxilin in order to evaluate the cytoarchitecture.

### Immunohistochemistry

Some sections from P9 and P23 animals were simultaneously immunoreacted by incubating them in a 50 mM Tris buffer (pH 7.4) containing 0.1% bovine serum albumin (BSA), 0.2% non fat milk powder and 0.3% Triton X-100 for one hour, followed by incubation with the monoclonal primary antibodies anti-microglial specific antigen (ED1 - 1∶200 – catalog number MB1435 - Chemicon) or anti-Glial Fibrillary Acidic Protein (GFAP - 1∶250 – catalog number G3893 - Sigma), diluted in the histochemistry reaction solution and maintained at 37°C under constant shaking for one hour. Then both reactions were stopped and, after two washes in PBS, sections were incubated with the Mouse ExtrAvidin-peroxidase staining kit (EXTRA2, Sigma), revealed using diaminobenzidine (DAB) as chromogen. Sections were washed twice in PBS and mounted in gelatinized slides, dehydrated and coverslipped.

Sections were analyzed under light microscopy using an Olympus BX 40 microscope. Image capturing was performed with a cooled-charged-coupled device camera (Olympus DP71). Gray scale images were taken using Adobe Photoshop software.

### Cell counts

Ten sections from each animal were photomicrographed (400× magnification) in one focal plane, including the WM in the vermis region, near deep cerebellar nuclei. Using the Image Pro Plus software, a 100 µm^2^ square was placed randomly, on each section of the WM region, and NADPH-d+ cells were quantified.

### Quantitative analysis

Data are compiled as means and standard errors of the means. The Kolmogorov-Smirnov one sample tests (K-S) was used to assess the normality of the distributions of the variable. Significance is assumed at the level of P<0.05. NADPH diaphorase positive cell numbers in the cerebellar white matter were compared between HI, SHAM and NM and were analyzed by means of two way analyses of variance (tANOVA). *AGE* and *TREATMENT* were considered the between-subjects factors. Lower-order ANOVAs and the Fisher's Protected Least Significant Difference (FPLSD) were used for post-hoc analyses.

## Results

### Qualitative analysis

We evaluated the pattern of NADPH-d staining, which presents a dark-blue colored product, in the vermis region of the cerebellum during postnatal development in NM, SHAM and HI animals. Both neuronal and glial alterations were observed regarding NADPH-d staining and distribution.

**Figure 1:**
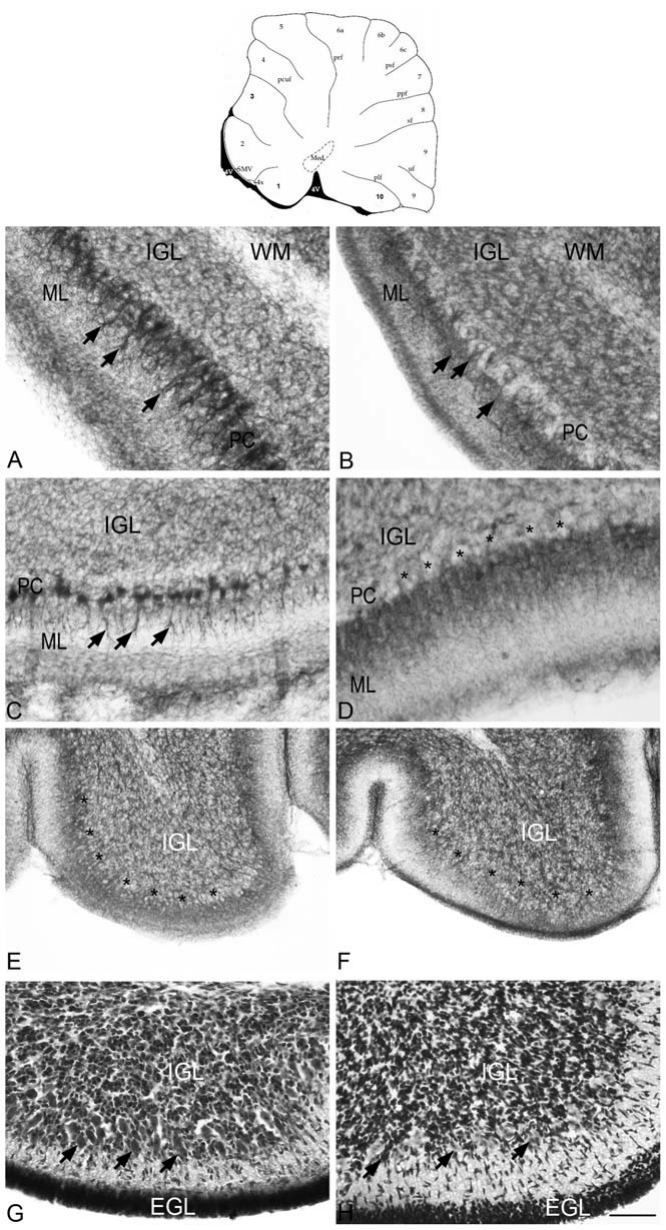
Pattern of NADPH-d labeling in Purkinje cells at P9 shown here after gray-scale conversion of the original color image. The scheme in the uppermost part of the figure indicates in bold and italic the folia shown in the figures A to H, at the vermis region (0.5 mm mediolateral distance). Med - medial (fastigial) cerebellar nucleus; Pcuf - preculminate fissure; Prf - primary fissure; Psf - posterior superior fissure; Ppf - prepyramidal fissure; Sf - secondary fissure; Uf - uvular fissure Plf - posterolateral fissure; ML - molecular layer. A, B: At folium 3 in SHAM animals (A), Purkinje cell bodies have a strong labeling and the dendritic trees are well defined by NADPH-d staining (arrows), while in HI animals (B), cell bodies have a weaker staining with a less defined staining pattern in the dendritic arbors (arrows). C, D: At folium 1 in SHAM animals (C), both cell bodies and processes have a strong labeling (arrowheads), while in HI ones (D), the cell bodies do not present NADPH-d activity (asterisks in the presumptive PC). Observe in D that the ML presents a strong and diffuse staining that corresponds to the location of the dendritic trees of the Purkinje cells. E, F: At folium 10, none of the groups presented NADPH-d staining in Purkinje cell bodies or in the dendritic arbors (asterisks in the presumptive PC). The presence of Purkinje cells was verified by hemaetoxilin staining in an adjacent section (G – SHAM; H – HI, arrows). WM = white matter; IGL = internal granular layer; EGL = external granular layer. Calibration bar: A–D, G and H = 50 µm, E and F - 100 µm.

**Figure 2:**
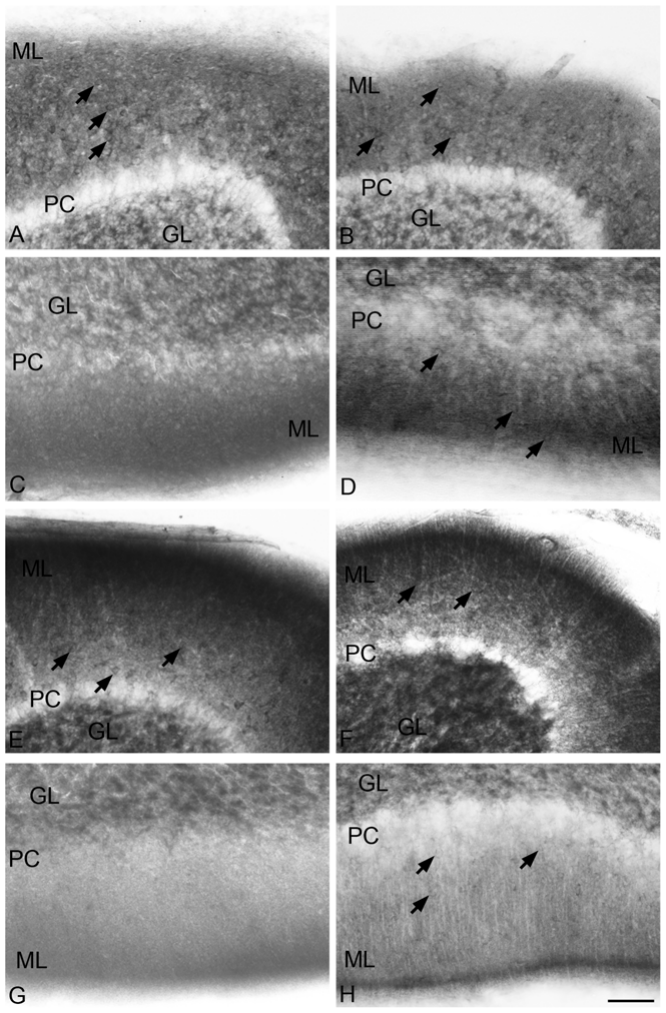
Distribution of NADPH-d stained cells, shown here in black after gray-scale conversion of the original color image, in the molecular layer (ML) of different folia in the vermis region (0.5 mm mediolateral distance) of the cerebellum at P23 and P90. A, C, E and G (SHAM); B, D, F and H (HI). A–D: P23 - A and B, folium 6; C and D, folium 10; E–H: P90 - E and F, folium 6; G and H, folium 10. At P23 and P90, SHAM animals presented a diffuse NADPH-d staining in the ML and a great number of NADPH-d+ cell bodies in folium 6 (A and E - arrows). This same pattern is observed in HI animals (B and F - arrows). In folium 10, no labeled cells were seen in the ML of SHAM animals (C and G), whereas HI animals presented a weaker staining in the ML in the same folium and ectopic NADPH-d+ cells near the PC, but also in the superficial ML (arrows in D and H). Granular cell layer showed a maintained NADPH-d staining in both groups. ML = molecular layer; PC = PC. Calibration bar: 50 µm.

At P9, all groups presented a weak NADPH-d staining in the EGL and a stronger staining in the IGL, with no apparent differences between groups. In the IGL, we observed a marked NADPH-d staining in cell bodies. Regarding Purkinje cell layer (PC), NM and SHAM animals, presented a strong staining in the Purkinje cell bodies in folia 1 to 7 (Figure 1A), and a weaker staining in folium 8. The dendritic arborization was also stained and individualized, as shown in figure 1C in a SHAM animal (arrowheads). In HI animals a weaker staining was seen in Purkinje cell bodies in folia 2 to 8 (Figure 1B). The dendritic trees were also not well defined in HI animals. In the folium 1 no staining was seen in Purkinje cell bodies at P9 (asterisks in figure 1D showing the presumptive PC), but a strong and diffuse staining was observed in the ML, where the dendritic arborization of Purkinje cells should be located. We did not observe NADPH-d staining in Purkinje cell bodies in folia 9 and 10 in any group (Figure 1E and F). To verify whether Purkinje cells were located in their usual place, hemaetoxilin stained sections were analyzed. The typical large and round Purkinje cell bodies were observed in all groups (Figure 1G and H – arrows). At later stages (P23 to P90) no NADPH-d staining was observed in Purkinje cell bodies, regardless of the experimental group (cf. Figure 2).

In the ML of folia 5 to 9 at later stages (P23 to P90), NM and SHAM animals presented a diffuse NADPH-d staining pattern with many stained cell bodies (showed in a SHAM animal in figure 2, A and E) that may correspond to basket and/or stellate cells. HI animals also presented the same pattern of staining in these folia (Figures 2, B and F). In the ML of folia 1 to 4 and 10, at these ages, no labeled cells were observed in NM and SHAM animals (Figure 2, C and G). HI animals presented an apparently weaker labeling in the ML at the same folia, with ectopic NADPH-d+ cells next to the PC, which corresponds to the location of basket cells, and also in the superficial ML (arrows in figure 2, D and H). Granular cell layer showed a maintained NADPH-d staining, which is similar in all groups. The cell bodies that were observed at this age in the GL were larger and stronger than those observed in P9 animals. Moreover, no difference was observed between the groups. Table 1 summarizes the major qualitative differences concerning NADPH-d distribution in Purkinje and molecular layers.

**Table 1:** Distribution of NADPH-d+ cells in the cerebellar gray matter.

NADPH-d distribution					
Purkinje cells			Molecular layer		
Postnatal day	NM/SHAM	HI	Postnatal day	NM/SHAM	HI
P9			P23 to P90		
folium 1	+++	0	folia 1–4	0	+/0
folia 2–7	+++	+	folia 5–9	++	++
folium 8	++	+	folium 10	0	+
folia 9–10	0	0			

Legend to the table: Summary of the major differences between groups in P9, and P23 to P90. Intensity of staining in cell bodies and dendritic trees are shown using a scale (+ to +++). 0 indicates the absence of stained cells. +/0 indicates that positive cells were no longer observed at P90.

**Figure 3:**
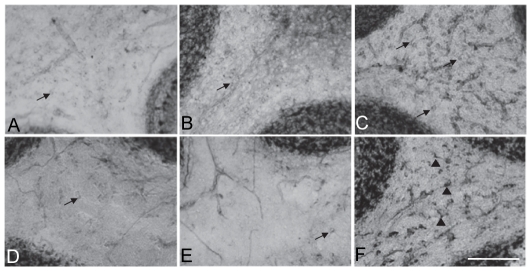
NADPH-d labeling in the cerebellar white matter (at the vermis region - 0.5 mm mediolateral distance) during development. A, B and C - P9; D, E and F - P23. A e D (NM); B and E (SHAM); C and F (HI). Observe that NADPH-d staining shows a higher number of cells (shown here in black - arrows) in HI animals, when compared to NM and SHAM ones. Observe in F that the morphology of the positive cells (arrowheads) in P23 HI animal is different from the NADPH-d stained cells in NM/SHAM animals at the same age and from all groups at P9. Calibration bar: 50 µm.

We also evaluated the pattern of NADPH-d distribution in the deep cerebellar white matter (WM), near to the cerebellar nuclei, in the vermis region. At P9, small rounded NADPH-d+ cells that present morphological features of microglia were observed in all groups (Figure 3A–C). At P23, in the HI group, a strong NADPH-d staining was observed in the cell bodies as well as in the processes of cells that were larger than those at P9. The morphological features of these NADPH-d+ cells were similar to reactive astrocytes. Small NADPH-d+ rounded cells were still present in NM/SHAM groups, although in a lower number when compared to the same groups at P9. At P45 and P90, we did not observe any NADPH-d+ cells in cerebellar WM in any of the groups.

**Figure 4:**
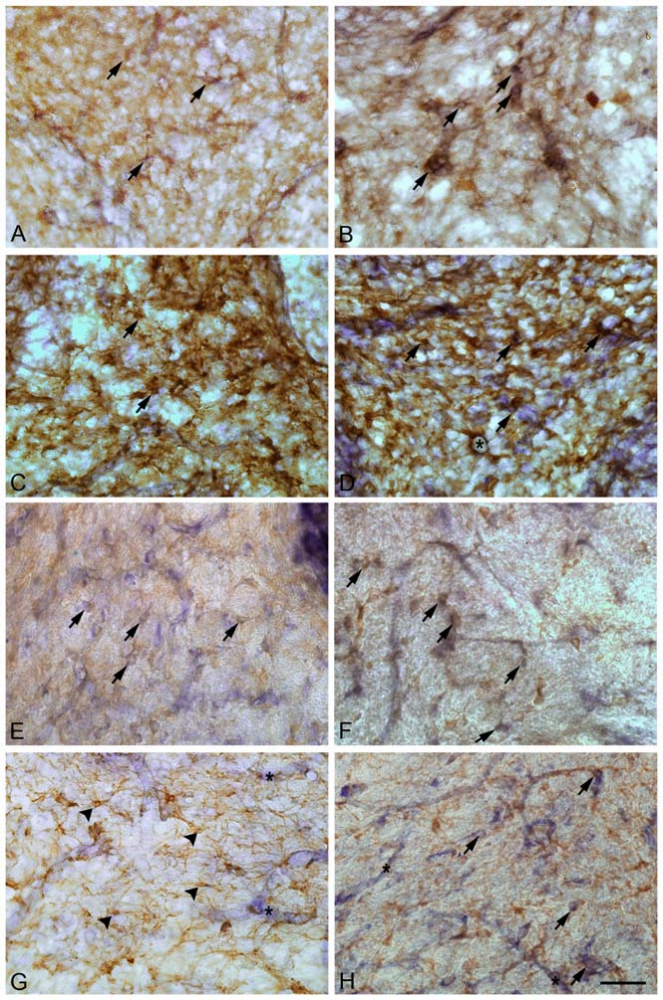
Double labeling with NADPH-d histochemistry (dark-blue) and microglia or astroglia immunoidentification (brown) in the vermis region of the cerebellar WM (0.5 mm mediolateral distance), during development. A–D - P9; E-H - P23. A, C, E and G (SHAM); B, D, F and H (HI); A–B and E–F – double labeled with anti-ED1 antibody; C–D and G–H – double labeled with anti-GFAP antibody. In both groups at P9, we can observe small, rounded NADPH-d+/ED1+ cells (A and B) or NADPH-d+/GFAP+ cells (C and D), as indicated by arrows. In D, observe a blood vessel, transversally cut, which presents NADPH-d staining, surrounded by GFAP+ astrocytic endfeet (asterisk). At P23, observe small rounded NADPH-d+/ED1+ cells in both groups, as indicated by arrows. HI animals display NADPH-d+/GFAP+ cells with typical reactive astrocyte morphology (arrows). SHAM animals do not present NADPH-d+/GFAP+ cells resembling reactive astrocytes. Arrowheads point to typical GFAP+ astrocytes, with no NADPH-d labeling. Notice the presence of NADPH-d+ blood vessels (asterisks in G and H) that are surrounded by GFAP+ astrocytic processes in HI animals (H) but not in SHAM animals (G). Calibration bar: 50 µm.

In order to identify NADPH-d+ cells in the WM, we also double labeled some sections with specific markers for microglia (ED1) or astrocytes (GFAP). At P9, both SH and HI animals presented NADPH-d+/ED1+ cells (Fig. 4A and B - arrows) and NADPH-d+/GFAP+ cells (Fig. 4C and D - arrows). In both groups the morphology of NADPH-d+/ED1+ cells are typical, i.e., small and rounded cells. At P23, SH and HI animals still presented NADPH-d+/ED1+ cells in the WM (Fig. 4E and F - arrows), with the same morphology as in P9. At P23, HI animals still presented NADPH-d+/GFAP+ cells similar to reactive astrocytes (Fig. 4H - arrows). However, SH animals did not present NADPH-d+/GFAP+ cells morphologically similar to reactive astrocytes, but instead showed typical GFAP+ astroglia (Fig. 4G - arrowheads).

### Quantitative analysis

The number of NADPH-d+ in the deep WM of the vermis region at P9 (14.3±1.3) was significantly higher than at P23 (11.0±1.0) (*AGE* effect: F = 7.8, df = 1, P = 0.01). We also observed that the HI procedure significantly affected the number of NADPH-d+ cells (*TREATMENT* effect: F = 12.8, df = 2, P<0.001): HI animals (16.7±1.1) had a significantly higher NADPH-d+ cell count than SHAM (11.6±1.5) and NM (9.7±0.7) ones (FPLSD: P<0.01 in both pairwise comparisons). No difference was observed between these last two groups.

**Figure 5:**
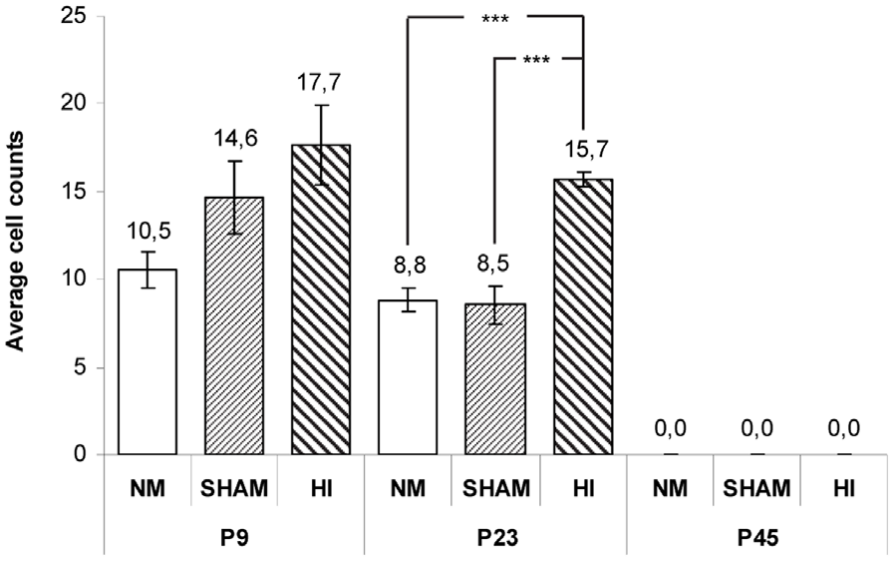
Average cell numbers per 100 µm^2^ in the cerebellar white matter of HI, SHAM and NM animals at P9, P23 and P45. 5 animals in each age from at least 3 different litters per group were used (maximum of 2 pups per dam). Note that, at P23, the number of cells in HI group is significantly higher than in the SHAM and NM and that no difference was observed between these last two groups. At P45 no NADPH-d+ cells were observed in all groups. *** P<0.001.

Given the aforementioned *AGE* effect, we decided to proceed with the analysis using lower-order ANOVAs for each age (P9 and P23) separately (Fig. 5), *TREATMENT* being used as the sole between-subjects factor. While the difference between P9 groups only approached difference (*TREATMENT* effect: F = 3.7, df = 2, P = 0.057), a significant difference between groups was observed for P23 animals (F = 27.2, df = 2, P<0.001): NADPH-d+ cell counts in HI animals were significantly higher than in SHAM and NM ones (FPLSD: P<0.01 in both pairwise comparisons). No difference was observed between these last two groups.

## Discussion

The cerebellum is the neural structure that produces the highest levels of NO within the CNS. These high levels of NO might make the cerebellum more susceptible to oxidative and nitrosative stresses, particularly in events such as HI. Our results have shown that a HI insult at E18 has a significant impact much latter on NOS expression within the critical period of cerebellum differentiation.

Many studies about NOS expression during cerebellar development have pointed that Purkinje and basket, stellate cells, as well as cells in the IGL, express NOS. However, most studies have not systematically described the cerebellar regions, perhaps assuming that the labeling would be uniform among them. However, Altman [27] have shown that each cerebellar folium has a different timing considering development. For example, the folium 10 is the first to finish proliferation and migration, whereas folia around the fissure prima develop more slowly, finishing their maturation around the end of third postnatal week.

About the time of insult, neurogenesis of Purkinje neurons is already finished. At birth they form a six cell thick layer, which, by day 3–4, is transformed into a single layer. The EGL already covers the entire cerebellum at birth. The IGL is formed by a migration of cells from the EGL that begins by day 7, with a peak at days 10–11, and is finished around 21 days [49]. Basket cells and stellate cells are formed at days 6–7 and days 10–11 respectively. Purkinje cell morphology changes from a spindle-shaped cell (P0) to the mature form with a sophisticated dendritic arbor, attained by the third postnatal week. This process requires intrinsic and extrinsic factors (for a review see [50]), such as electrical activity and glutamate, which is known to promote the dendritic differentiation of Purkinje cells [51], [52]. Expression of NOS by Purkinje cells is transient in rats, occurring only around the second post-natal week, and is highly correlated with the migration period and with the presence of the EGL [53], [54]. In accordance with this observation, we also showed a transient NADPH-d staining in Purkinje cells in the present study.

At P9, in control animals, Purkinje neurons showed a strong NADPH-d staining in cell bodies and also in well-developed dendritic trees in most cerebellar folia, whereas, in HI animals, the staining in Purkinje cell bodies was weaker and labeling in their dendritic trees revealed a less developed arborization. Purkinje cells from folium 1 in HI animalsat P9 did not present NADPH-d staining in their cell bodies, but an intense staining appeared in the dendritic arborization located in the molecular layer, while NM and SHAM animals presented staining both in the cell body and in their processes. The HI insult could alter the normal development of the EGL by an increase in cell death, which could be associated with changes in the microenvironment, such as increases in inflammatory cytokines and free radical levels, as already shown in the cerebral cortex by Robinson and colleagues [40]. The higher levels of inflammatory cytokines as well as the increase in lactate levels in cerebellar neuroepithelia after HI insult [46] could lead to an increase in cellular death in the EGL, and the resulting inflammation could provoke the translocation and hyper-stimulation of NOS in the dendritic trees, distributing the enzyme to the place of higher activity.

It has been shown by Pisu and colleagues [29] that Purkinje cell bodies from P10 animals that received a single injection of cisplatin, an anti-proliferative drug, present a reduction in the expression of NOS and NADPH-d activity. Moreover, they also described that, in the same period, the ionotropic receptor GluR2 evidenced a less developed dendrite of Purkinje neurons in the top of the lobules, demonstrating that by affecting cell proliferation at this time (mostly of granular cells precursors), the differentiation of Purkinje cells was impaired. Other studies have shown an increase in the NADPH-d activity after chemical, thermal and mechanical lesions [55]–[57]. Tenorio and colleagues [58] observed an impairment in the distribution of NADPH-d activity in retinorecipient cells of the superior colliculus in rats that had an eye enucleated in early development; although the cellular morphology was not changed, there was a restriction of NADPH-d staining to cell bodies, which demonstrated that the enzyme was no longer distributed to distal dendrites after the lesion. All together, these works show that the expression of NADPH-d activity may be affected differently according to the insult.

We did not see any gross difference in the pattern of NADPH-d staining in either the external or the internal granular layer at P9, as well as in the granular layer at P23 and P90. To our knowledge, there is no data concerning NADPH-d/NOS distribution in granule cells in HI models. However, concerning the vulnerability of the granular cells to HI, apoptosis has been reported in animals submitted to focal cerebral hypoxic–ischemic insult at P7 [59]. In another study comparing a focal cerebral-hypoxic ischemic insult at P2 with hypoxia alone at the same age [60], a significant reduction was observed at P21 in the thickness of the molecular and granular layers in parallel with a significant decline in the number of Purkinje cells in the different lobules of the cerebellum in hypoxic as well as in hypoxic-ischemic animals when compared to sham, ones. A proliferation deficit of external granule cells and Purkinje cell loss have also been reported in the fetal sheep following prenatal global hypoxia, although the area of the IGL was not impaired [61]. These data suggest that, depending on the time of HI insult, the granule cells may respond differently. Besides, in none of these works the animals were submitted to a global systemic HI insult as in present study.

In the molecular layer, ectopic NADPH-d+ cells were observed in folia 1 to 4 and folium10 only in HI animals at P23. At P90, in HI animals, the folia 10 still had ectopic NADPH-d+ cells in the ML. Since migration from EGL to IGL requires that Purkinje cells produce a NO gradient [54] we could hypothesize that the NADPH-d+ cells in ML are granular cells that had their migration towards IGL disrupted. Data about the role of NO in cell migration has been described in several studies [54], [62]. Inhibition of nNOS significantly decreased the migratory index of granule cells in slice cultures from postnatal rat cerebellum [54]. Cell proliferation and migration were significantly increased in the adult subventricular zone (SVZ) and in the dentate gyrus when a NO donor was added in normal and hypoxic animals [63]. Cajal-Retzius cells are nNOS-positive until birth, when the cortical migration ends, suggesting an important role of NO in this process [62]. Conversely, as we were not able to distinguish the phenotype of these cells based on their morphology, we cannot discard the possibility that basket and/or stellate cells might have also increased NADPH-d after HI insult in folium 10.

Multiple types of injury resulting from preterm birth in humans, including systemic HI, converge to hinder neural cell survival, particularly for immature oligodendrocytes and cerebral neurons [64]. Impaired neural cell survival and differentiation continues for a prolonged period after the initial injury [40], [47]. During the time of the HI insult, as well as in the days following the insult, when the levels of cytokine and other inflammatory modulators are still elevated [40], several glial and neuronal progenitor populations are entering the cerebellum parenchyma through the prospective cerebellar white matter (WM). These progenitors, especially of oligodendrocytes, are more vulnerable to HI events because they lack the enzymatic complexes capable of dealing with the great amount of free radicals produced during HI. NO could form free radicals if produced in large amounts, and it could be toxic to oligodendrocyte progenitors. Other than generation of free radicals, a number of pathways such as N-methyl-D-aspartate (NMDA)-mediated intracellular Ca^++^-influx and Calcium/cyclic AMP response-element binding protein (CREB)-mediated transcription of apoptotic proteins such as Bax, Bad and Bcl-xl are triggered by NO resulting in neuronal death [65]–[68]. Increased expression of Bax but not Bcl-2 in hypoxic cerebral tissue, thus increasing the Bax/Bcl-2 ratio in favor of hypoxic-induced apoptosis, has been reported [69]. NO induces the proapoptotic cascade by increasing phosphorylation of Bcl-2 by inactivation of mitogen-activated protein kinase (MAPK) phosphatases and consequent activation of extracellular signal-regulated kinase (ERK) and c-Jun N-terminal kinase (JNK) [69]. Other mechanisms by which NO contributes to cytotoxicity may be peroxynitrite-mediated oxidative damage, DNA damage and energy failure [70]–[73].

At 9 days, a significantly greater number of small rounded NADPH-d+ cells were seen in deep cerebellar WM. These cells appeared to be microglia, based on the analysis of size and phenotype. This classification was confirmed with double labeling with anti-ED1 antibody. Microglia are known to produce a great amount of NO through iNOS, and this NO could be responsible for some alterations observed in oligodendroglial cells in several studies [74], [75], [32]. At P23, few NADPH-d+ cells were seen in NM/SHAM animals. Labeling was weaker than at P9. HI animals, however, still had a large number of cells, now with a reactive astrocyte morphology, presenting NADPH-d labeling in both cell body and in the processes. NADPH-d+/GFAP+ cells were found in deep cerebellar WM at this age only in HI animals. This suggests that the HI insult leads to a sustained astrogliosis that can be observed even four weeks after the event. NO being released during this critical period of development could lead to an increase in oligodendrocyte progenitor loss and migration, which was still present at this age [76]. In fact, we also observed a failure in the differentiation of oligodendroglia in cerebellum of HI animal (unpublished data).

Overall, our results show that prenatal HI affects the pattern of NADPH-d expression/distribution in the cerebellum. An increase in NADPH-d expression is detected in the deep cerebellar WM, whereas redistribution is observed in the PC and in the ML. Regarding the PC, we observed a decrease in labeling in Purkinje cell bodies and a redistribution of the enzyme close to primary dendrites in HI animals. Apparently, these different responses to hypoxia are related to the different functions NOS exerts in glia and neurons. Within the molecular layer, these changes could lead to alterations in cytoarchitecture, probably by interfering in migration events and synaptic refinement, which could be contributing in a determinant manner to the deficits in cerebellar function.

Furthermore, NADPH-d reactive microglia and astrocytes in the deep WM during a critical period of oligodendroglial differentiation may also contribute to the delay in motor development observed by Robinson and colleagues [38] using this model of HI. This rodent HI insult may mimic human prenatal brain injury in several important ways and could provide novel insights into the pathogenesis of human perinatal brain insults.
